# Novel Pathogenic Mucorales Identified Using the Silkworm Infection Model

**DOI:** 10.3390/jof7110995

**Published:** 2021-11-22

**Authors:** Suresh Panthee, Hiroshi Hamamoto, Yayoi Nishiyama, Atmika Paudel, Kazuhisa Sekimizu

**Affiliations:** 1 Drug Discoveries by Silkworm Models, Faculty of Pharma-Science, Teikyo University, Hachioji, Tokyo 192-0395, Japan; supanthee@main.teikyo-u.ac.jp; 2 Institute of Medical Mycology, Teikyo University, Hachioji, Tokyo 192-0395, Japan; hamamotoh@main.teikyo-u.ac.jp (H.H.); ynishiya@main.teikyo-u.ac.jp (Y.N.); 3 International Institute for Zoonosis Control, Hokkaido University, Sapporo, Hokkaido 001-0020, Japan; atmikapd@czc.hokudai.ac.jp

**Keywords:** Mucorales, COVID-19, opportunistic infection, silkworm, animal-model

## Abstract

Mucormycosis, a rare but highly fatal infection, is caused by fungi of the order Mucorales. Due to their ubiquitous nature, reduced susceptibility to antifungals, acid tolerance, and ability to infect immunocompromised patients through rapid dissemination, these fungi have been frequently reported to infect the COVID-19 patients. In order to develop strategies to overcome mucormycosis, it is essential to understand and identify novel Mucorales present in the environment. In this study, we report the identification of four novel pathogenic Mucorales using the silkworm (*Bombyx mori*) model. The strains’ phylogeny was analyzed using the genome sequence of the large subunit ribosomal ribonucleic acid (LSU rRNA) and the internal transcribed spacer (ITS) region, where strains 1-3, 5-3, and S286-1101 claded with *Mucor orantomantidis*, and strain 827-14 claded with *Backusella lamprospora*. All the strains had a cold-sensitive phenotype with their inability to grow prominently at 4 °C. *Mucor* sp. 1-3 and 5-3 were characterized by their filamentous and yeast-like growth under aerobic and anaerobic conditions, respectively. The yeast colonies of *Mucor* sp. 5-3 had multipolar budding cells often observed with cleaved cell surfaces under a scanning electron microscope. We further found that these strains were able to kill immunocompromised mice suggesting their pathogenicity to mammals. Our study established an invertebrate model-based screening system to identify novel pathogenic Mucorales from the natural environment and provided a clue towards the rapid increase in COVID-19 related mucormycosis.

## 1. Introduction

Fungi help maintain the diversity of the ecosystem and are critical to nutrient-cycling by degrading dead organic materials [1]. In nature, fungi with agricultural, ecological, economic, biotechnological, and medical importance have been identified. On the other hand, fungi can also cause human infections. Invasive fungal diseases account for a majority of complications among immunocompromised patients worldwide [2]. Fungi of the order Mucorales cause mucormycosis, a rare but highly fatal fungal infection. They can cause cutaneous, rhino-orbital, pulmonary, rhino-cerebral, and disseminated bloodstream infections; the severity and prognosis largely depend upon the infection type. The fatality rate is very high [3] with 96% among patients with disseminated infections [4]. The incidence of mucormycosis is rapidly increasing, especially in developing countries like India and Nepal, where Mucorales were mostly found to cause rhino-orbito-cerebral infections. The most recent incidences are observed in COVID-19 patients [5,6,7] or those who recently recovered from COVID-19. Given that the incidence of mucormycosis has been associated with preexisting conditions such as uncontrolled diabetes mellitus, malignancies, trauma, or extended corticosteroids use [4], it is likely that the number of cases with mucormycosis will increase further due to the current COVID-19 pandemic. Primarily found in soil and decaying vegetation, Mucorales are ubiquitous, have reduced susceptibility to most clinically used antifungal agents, and thrive under high acid conditions. Their thermotolerance, however, is greatly varying; some, such as *Rhizopus microspores* thrive at temperatures as high as 50 °C [8], while some, such as *R. sexualis* cannot grow above 25 °C [8]. Based on their thermotolerance, it was previously thought that Mucorales that cannot grow at 37 °C were medically not important. However, case reports and incidences of cutaneous infections caused by *M. hiemalis* [9,10,11,12], which cannot grow at 37 °C [13,14], have been observed. These cases of infections suggested that the fungi that do not grow at 37 °C are capable of causing superficial infections. Furthermore, invasive mucormycosis rapidly disseminates within the host tissues. Depending upon the infection site, Mucorales interact with specific host receptors and take advantage of the host physiological conditions to derive nutrients such as iron [15]. Furthermore, Mucorales can store iron in ferritins besides siderophores, further depleting the host of iron and proliferating within the host rapidly [16]. Therefore, patients with diabetes mellitus and COVID-19 are more prone to mucormycosis as these patients have a higher level of free iron in their blood [17,18]. As mucormycosis has been increasingly common, studies on the understanding of their pathogenesis as well as identifying emerging virulent strains is of top priority.

In our routine investigation of the environmental mycobiome using the silkworm (*Bombyx mori*) model, we isolated fungi with morphological features similar to Mucorales. In this manuscript, we report the detailed molecular phylogenic and morphological analysis of four novel strains identified from plant sources.

## 2. Materials and Methods

### 2.1. Isolation and Growth of Fungal Strains

Fungal strains were isolated from plant seeds, dead and live plant leaves, and living plant buds collected from Chiba and Saitama, Japan. The collected samples were stored in sterile falcon tubes at 4 °C until used for analysis. The samples were washed with water and resuspended in normal saline. Serial dilutions were then spread onto YPD agar plates (yeast extract 10 g/L, tryptone 20 g/L, glucose 20 g/L, sodium propionate 2 g/L, and agar 15 g/L) supplemented with 100 µg/mL chloramphenicol. The plates were incubated at 30 °C overnight. Single colonies were selected, and pure cultures were regrown on YPD agar plates at 30 °C. For the preparation of the spores, the pure culture of the strains was spread onto PDA (Potato Starch (from infusion) 4 g/L, dextrose 20 g/L, agar 15 g/L) plate and incubated at 30 °C for 2–5 days. After sporulation, the spores were then resuspended in normal saline containing 0.05% Tween 80 and filtered through a 40 µm cell strainer attached to a 50 mL falcon tube. The spores were washed again with normal saline, counted using a C-Chip, and stored at 4 °C until use.

### 2.2. Morphological Studies

For growth analysis, 4.0 × 10^4^ spores contained in 40 µL of normal saline were spotted at the center of the YPD agar plate. The plates were allowed to dry under a clean bench and incubated at different temperatures of 37 °C, 30 °C, and 4 °C. The growth status was recorded at different time intervals. The strains were incubated using a sealed rectangular jar containing AnaeroPack (Mitsubishi Gas Chemical, Tokyo, Japan) to examine the anaerobic growth.

We adopted the slide culture technique for morphologic analysis of the fungi, followed by the observation under a light microscope (BX53, Olympus, Tokyo, Japan). Fresh fungal spores prepared on PDA were inoculated on a PDA agar block (ca 10 mm × 10 mm) contained in a sterile glass slide. The agar block was then covered with a sterile coverslip and transferred to a sterile Petri dish containing sterile water and incubated at 30 °C. On day 4, fungal growth was observed, and morphological features were recorded using a microscope.

### 2.3. PCR Amplification, Sequencing, and Phylogenetic Analysis

Two loci: large subunit ribosomal RNA (LSU rRNA) and internal transcribed spacer region (ITS), were used for the phylogenetic placement of the fungal strains. Fragments of the LSU rRNA gene and ITS region of the fungal strains were amplified using PCR primer pairs 28SF1: 5′-AAGCATATCAATAAGCGGAGG-3′ 635: 5′-GGTCCGTGTTTCAAGACGG-3′, and ITS1F 5′-GTAACAAGGT(T/C)TCCGT-3′ ITS1R 5′-CGTTCTTCATCGATG-3′, respectively. The PCR products were then purified and sequenced on the 3130 Genetic Analyzer (Applied Biosystems, Foster City, CA, USA) using the same primers. The sequence of the ITS region of *Mucor* sp. 5-3 was obtained from the whole genome assembly. To perform evolutionary analysis, the LSU rRNA and ITS region sequences of the TYPE fungal strains were downloaded from NCBI, aligned using MUSCLE, and the evolutionary distances, in the units of the number of base substitutions per site, were computed using the Maximum Composite Likelihood method in MEGA X [19]. The reliability of internal branches was assessed using 500 bootstrap replications.

### 2.4. Genomic DNA Isolation, Whole Genome Sequencing, and Assembly

To isolate genomic DNA, *Mucor* sp. 5-3 was grown with aeration on 5-mL YPD medium contained in a 50-mL falcon tube using a shaker maintained at 30 °C. To 0.5 mL of full growth, 0.5 mL of zirconium-coated beads (Yasui Kikai YZB05, ϕ 0.5 mm, Osaka, Japan) were added, and the supernatant was removed after centrifugation. Cells were then lysed by vigorous agitation at 2400 RPM for 2 min using a multi-bead shocker MB455GU(S) (Yasui Kikai). The genomic DNA from the lysate was then purified using a DNeasy plant mini kit (Qiagen, Hilden, Germany). Next, the draft genome sequence of *Mucor* sp. 5-3 was prepared using the Ion PGM System (ThermoFisher Scientific, Waltham, MA, USA) as previously described [20,21]. Briefly, after confirming the quality and quantity of genomic DNA using nanodrop, agarose gel electrophoresis, and Qubit, the barcoded library of 400 base reads was prepared after fragmentation of 100 ng of the DNA using the manufacturer’s recommended protocol of the Ion Xpress™ Plus Fragment Library Kit (ThermoFisher Scientific). The prepared library was then enriched in an Ion 318™ Chip v2 using Ion Chef (ThermoFisher Scientific) and sequenced to obtain 5 M reads (average length 270 bp). The reads were then trimmed, and de novo assembled using CLC Genomics Workbench ver. 20.0.4 (CLC bio, Aarhus, Denmark).

### 2.5. Scanning Electron Microscopy 

The cell morphology of *Mucor* sp. 5-3 grown in aerobic and anaerobic cultures on YPD agar plate was observed using a high-resolution field emission SEM system, JSM-7500F (JEOL, Tokyo, Japan). First, the cells were fixed with 2.5% glutaraldehyde and postfixed with 1% osmium tetroxide. Afterward, the samples were dehydrated with graded acetone, freeze-dried in *t*-butyl alcohol, and coated with osmium tetroxide using an osmium plasma coater OPC 60A (Filgen, Nagoya, Japan). Specimens were then observed under JSM-7500F at an acceleration voltage of 1 kV.

### 2.6. Silkworm Pathogenicity Studies

Silkworm rearing was performed as previously explained [9]. Pathogenicity studies of the spore suspensions were performed using fifth instar day 2 larvae. For the initial pathogenicity test, the undiluted and one-eighth diluted spore suspension was injected into the hemolymph of three silkworms, incubated at 27 °C, and survival was recorded at 20 h. Next, for the comprehensive evaluation of pathogenicity, the spore suspensions were diluted using normal saline to a range of 5.0 × 10^6^–2.1 × 10^4^ spores/mL, and 50 µL of each dilution was injected into the hemolymph of 5 silkworms, and the larvae were incubated at 27 °C. The survival of the silkworms was examined at different time intervals, and the survival curve was plotted.

### 2.7. Mouse Pathogenicity Studies

All mouse experimental protocols were approved by the Animal Use Committee at the Genome Pharmaceuticals Institute. Four-week-old, female ICR mice were caged in a group of five in a cage and kept in a room maintained with a 12 h light/12 h dark cycle, 23–24 °C, and 55% humidity. Mice were immunocompromised with the intraperitoneal injection of 200 mg/kg cyclophosphamide at −2, +3, and +6 days relative to infection. Mice (*n* = 5) were intraperitoneally infected with spores equivalent to ca. 2.0 × 10^8^ colony forming units contained in 0.2 mL normal saline. Mouse survival was recorded at different time intervals, and the survival curve was plotted.

## 3. Results 

### 3.1. Taxonomy and Phylogenetic Analysis

**Figure 1:**
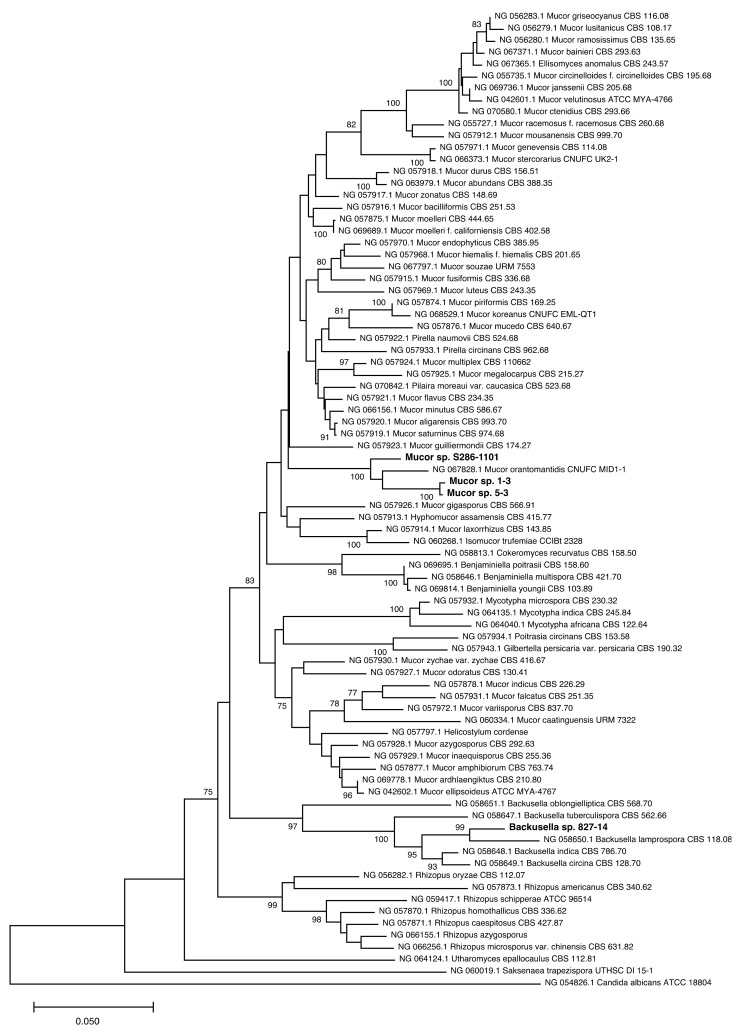
LSU rRNA-based phylogenetic analysis of Mucorales. The LSU rRNA sequence was utilized to infer the evolutionary relationship of the strains. The optimal tree, drawn to scale, with branch lengths in the same units as those of evolutionary distances, with the sum of branch length = 2.44139183, is shown. The percentage of replicate trees in which the associated taxa clustered together in the bootstrap test (500 replicates) are shown next to the branches. After the ambiguous positions were removed for each sequence pair, there were a total of 1552 positions in the final dataset. The novel strains are written in boldface with a larger font size. Sequence alignment of the four strains identified is presented in Supplementary Figure S2.

**Figure 2:**
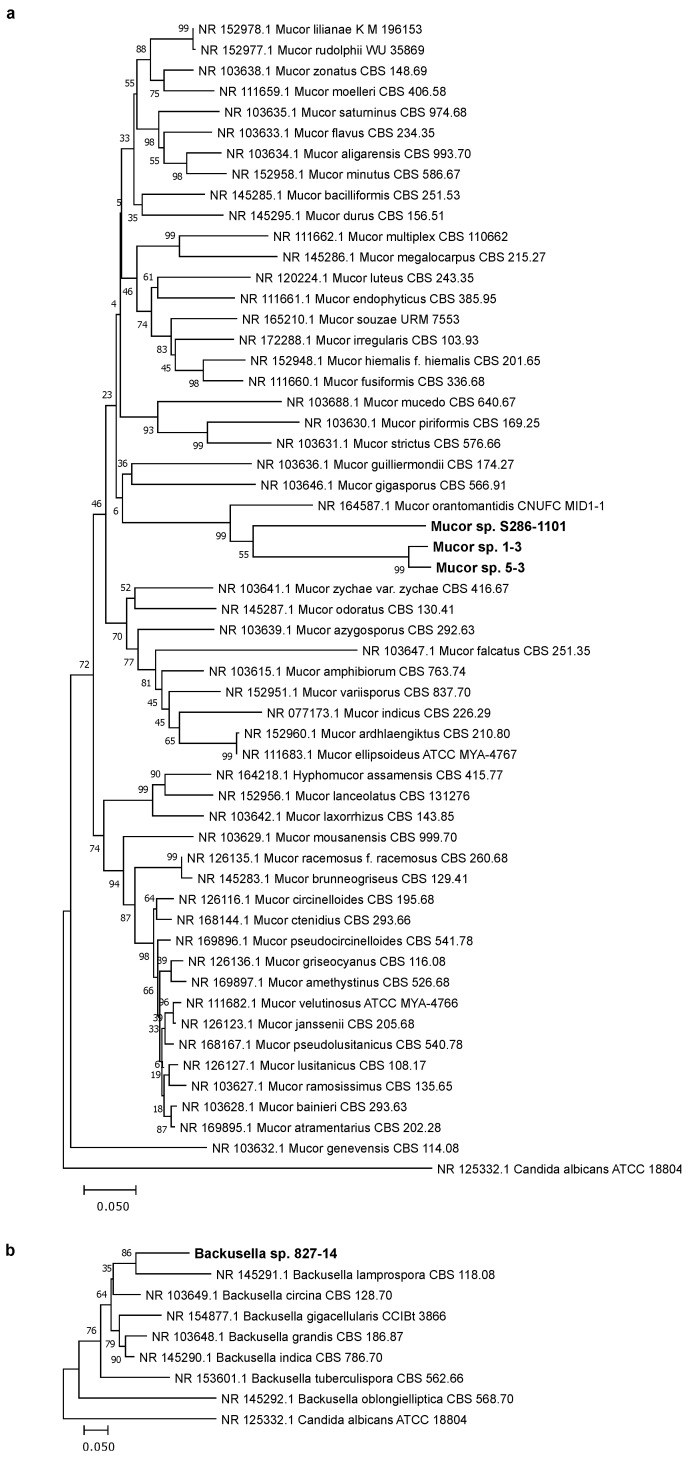
ITS-based phylogenetic analysis of Mucorales. The ITS sequence was utilized to infer the evolutionary relationship of the strains. The optimal tree, drawn to scale, with branch lengths in the same units as those of the evolutionary distances, with the sum of branch lengths of (**a**) 4.16205042 and (**b**) 1.37992789 for *Mucor* and *Backusella*, respectively, is shown. The percentage of replicate trees in which the associated taxa clustered together in the bootstrap test (500 replicates) are shown next to the branches. After the ambiguous positions were removed for each sequence pair, there were a total of 1064 (**a**) and 975 (**b**) positions in the final dataset. The novel strains are written in boldface with a larger font size.

We isolated four fungal strains from plant sources and attempted to identify them (Table 1). BLAST analysis of the LSU rRNA region showed that strains 1-3, 5-3, and S286-1101 were related to *Mucor orantomantidis* (accession no: NG_067828.1) with the percentage identity of 95.4% (641/672 bp), 95.4% (641/672 bp), and 97.2% (651/670 bp), respectively. Strain 827-14 was 96% (624/650) identical to *Backusella lamprospora* CBS 118.08 (accession no: NG_058650.1). This result was consistent with the constructed phylogenetic tree using MEGA X [19], where we found that strains 1-3, 5-3, and S286-1101 were claded with *M. orantomantidis*, and strain 827-14 was claded with *B. lamprospora* (Figure 1). These findings suggested that these strains were novel species belonging to *Mucor* (1-3, 5-3, and S286-1101) and *Backusella* (827-14). The taxonomic assignment of these strains was further confirmed by the phylogenic tree using ITS sequences, another locus used frequently for the taxonomic demarcation of Mucorales [13]. ITS analysis showed that *Mucor* strains were claded with *M. orantomantidis* (Figure 2a) and *Backusella* sp. 827-14 claded with *B. lamprospora* (Figure 2b). The phylogenetic tree was reconstructed using MAFFT [22] and RAxML [23] to reveal similar results (Supplementary Figure S1a–c). Among four strains, we randomly selected *Mucor* sp. 5-3 for whole genome sequencing and SEM analysis.

### 3.2. Analysis of the Mucor Genome Assembly 

We have previously used the next-generation sequencing tool for the genomic analysis of various bacteria [21,24] and fungi [20] and have found that compared to the bacterial genome, the fungal genome contains a large number of repeats and is difficult to assemble. We sequenced the *Mucor* sp. 5-3 genome using the Ion PGM System and found that its genome size was 30.8 Mb in size, had a G+C content of 39.26%, and divided into about 17,079 contigs (Table 2). This indicated a presence of a large number of repeat elements in the genome. Of note, the same sequencing approach, used for *Candida albicans* TIMM1768 resulted in about 3400 contigs for a 14.5 Mb genome [20]. Besides, combined with a previous study [25], it can be expected that the *Mucor* genome is diverse with a genome size ranging between 30–47 Mb.

### 3.3. Morphological Studies

**Figure 3:**
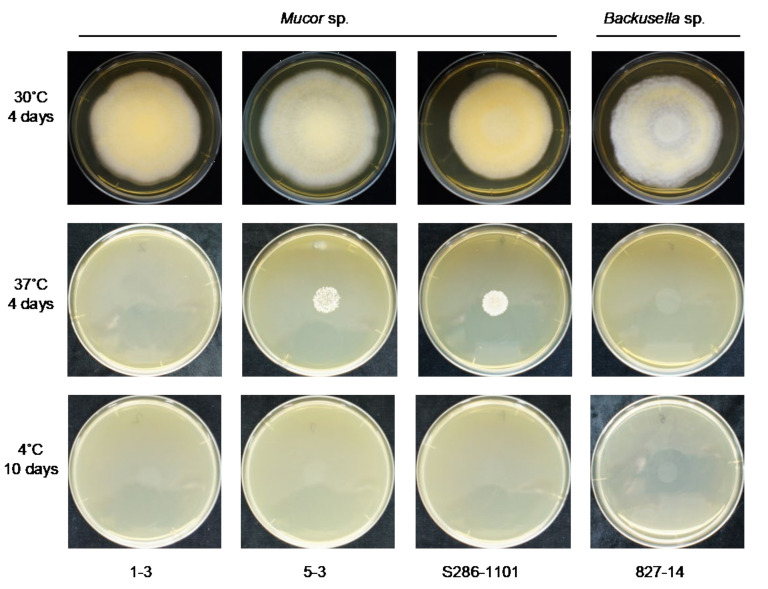
Temperature-specific growth of novel Mucorales. 4.0 × 10^4^ spores contained in 40 µL of normal saline were spotted at the center of the YPD agar plates, dried, and incubated at different temperatures for the designated duration before taking a picture. Everyday pictures of the plates incubated at 30 °C are included in Supplementary Figure S3.

**Figure 4:**
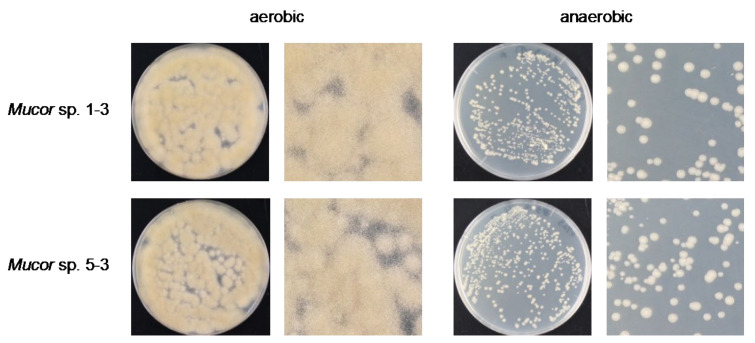
Aerobic and anaerobic growth of *Mucor* sp. 1-3 and 5-3.

**Figure 5:**
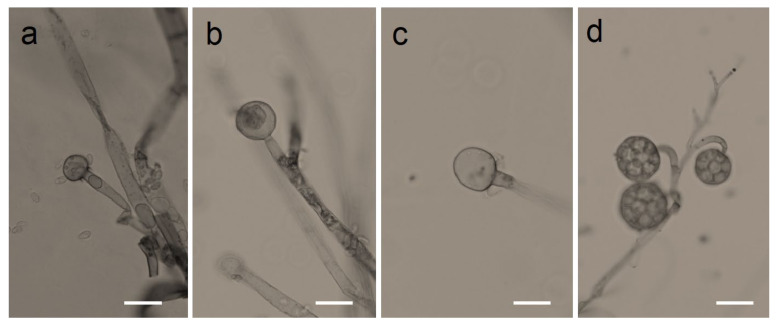
Photomicrographs of Mucorales grown on slide culture. *Mucor* sp. 1-3 (**a**), 5-3 (**b**), S286-1101 (**c**), and *Backusella* sp. 827-14 (**d**). Mucorales were grown aerobically for 4 days. Bars = 20 µm.

We found that all the strains grew well on the YPD agar plate at 30 °C when grown under aerobic conditions. However, they had a difference in their growth status at 4 °C and 37 °C. *Mucor* sp. 5-3, and S286-1101 could grow, although slowly, while the other two strains could not grow at 37 °C. At 4 °C, *Mucor* spp. did not show a visible sign of growth; however, *Backusella* showed a faint growth (Figure 3, Supplementary Figure S3). Next, we used *Mucor* sp. 1-3 and *Mucor* sp. 5-3 to check their ability to grow under anaerobic conditions. Incubation for two days at 30 °C after streaking on YPD agar showed that the cells grew as yeast under anaerobic conditions; however, mycelial growth was observed under aerobic conditions (Figure 4). Besides, we found that the colony morphology—when visualized with the naked eye—of these two strains on agar plates was similar. When sporangia were observed under a light microscope, a difference was observed among *Mucor* spp. and *Backusella* spp. (Figure 5a–d).

### 3.4. Mucor Morphology under High Resolution

**Figure 6:**
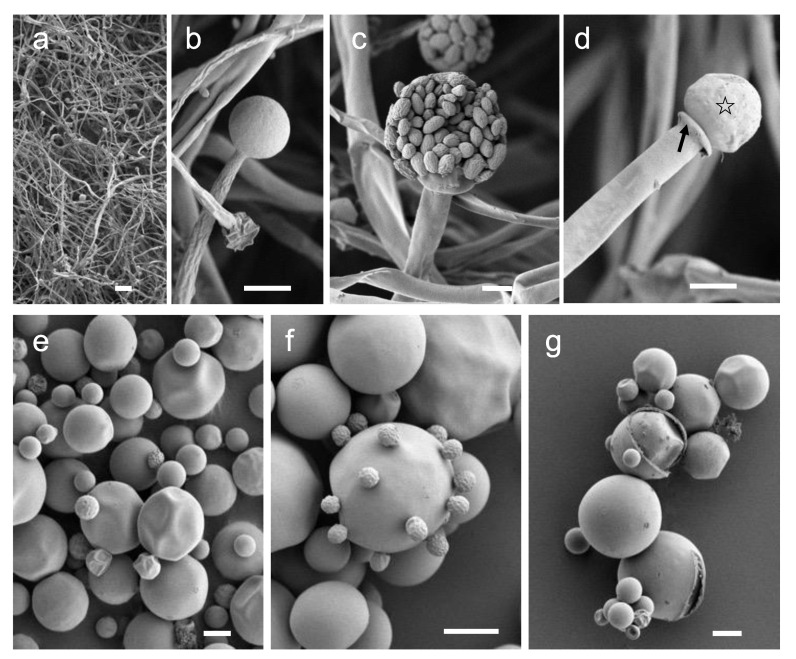
SEM images of aerobic (**a**–**d**) and anaerobic (**e**–**g**) culture of *Mucor* sp. 5-3. (**a**) Low magnification image of mycelium; aseptate hyphae and sporangia. (**b**) Sporangia formed at the tip of the sporangiophore. (**c**) Sporangiospores inside the sporangia. (**d**) Columella (open star) and collarette (filled arrow) were observed at the tip of the sporangiophore. (**e**) Yeast-like colonies are composed of spherical cells of various sizes. (**f**) Multipolar budding found in large cells. (**g**) Cleavage of surface structure is observed in large cells. *Mucor* sp. 5-3 was grown aerobically (**a**–**d**) and anaerobically (**e**–**g**) on a YPD agar plate for 2 days. Bars: (**a**) = 100 µm, (**b**–**g**) = 10 µm.

Using *Mucor* sp. 5-3, cell morphology of mycelial growth under aerobic conditions and yeast growth under anaerobic conditions were studied by Scanning Electron Microscope (SEM). Aerobic culture resulted in hyphal growth, where long elongated aseptate hyphae and sporangium were observed (Figure 6a,b). Inside the matured sporangium, a large number of sporangiospores were observed (Figure 6c). The tip of sporangiophore harbored columella and collarette (Figure 6d). The yeast colonies formed under anaerobic growth were made up of spherical cells of various sizes ranging from 2 to 30 µm in diameter (Figure 6e). Multipolar budding cells were observed in large cells (Figure 6e,f). Some budding cells had smooth surfaces, whereas some had small golf ball-like shapes (Figure 6f). Furthermore, strange cells with cleaved cell surfaces were often observed in large cells (Figure 6g). The existence of cells with cleaved surface layers has never been reported before. The significance of these cells in the growth of yeast cells needs to be investigated in more detail in the future.

### 3.5. Pathogenicity of Newly Isolated Mucorales 

**Figure 7:**
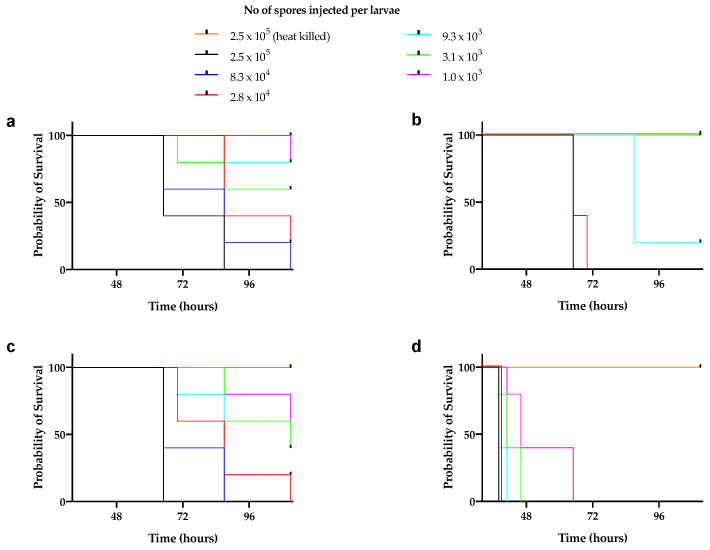
Pathogenicity of Mucorales spores in silkworm. Fifty microliters serially diluted spore suspensions of *Mucor* sp. 1-3 (**a**), *Mucor* sp. 5-3 (**b**), *Mucor* sp. S286-1101 (**c**), and *Backusella* sp. 827-14 (**d**) were injected into the silkworm hemolymph (n = 5). Silkworms were incubated at 27 °C and survival was recorded at various time points.

Although various vertebrates and invertebrates are used as animal models to study mucormycosis [26], silkworms were not used to test the pathogenicity of mucoralean fungi so far. Given that the use of the silkworm infection model for the test of pathogenicity of several bacteria and fungi is well established [27,28,29,30], we aimed to evaluate the pathogenicity of novel Mucorales using a silkworm infection model. First, we prepared a suspension of spores and examined the pathogenicity of the suspension without dilution. We found that all silkworms died at 20 h (Supplementary Table S1). Next, we prepared a 1/8—fold dilution of spore suspension, injected it into the silkworm, and checked the survival at 15 and 20 h. We found that at 15 h, most of the silkworms were surviving; however, the lethality of these Mucorales was high at 20 h, indicating the ability of these fungi to rapidly kill silkworms (Supplementary Table S1). These results indicated the pathogenicity of the spores of newly identified fungi against silkworms. Next, for a quantitative evaluation of pathogenicity, we serially diluted the spores and injected them into the silkworm hemolymph so that each silkworm received 2.5 × 10^5^–1.0 × 10^3^ spores. To confirm the establishment of infection, silkworms were inoculated with 2.5 × 10^5^ heat-killed spores of each strain. Whereas the heat-killed spores were nonpathogenic to silkworms, a dose-dependent killing by live spores was observed (Figure 7a–d). We found that with the same dose injected, *Backusella* were more pathogenic compared to *Mucor*.

**Figure 8:**
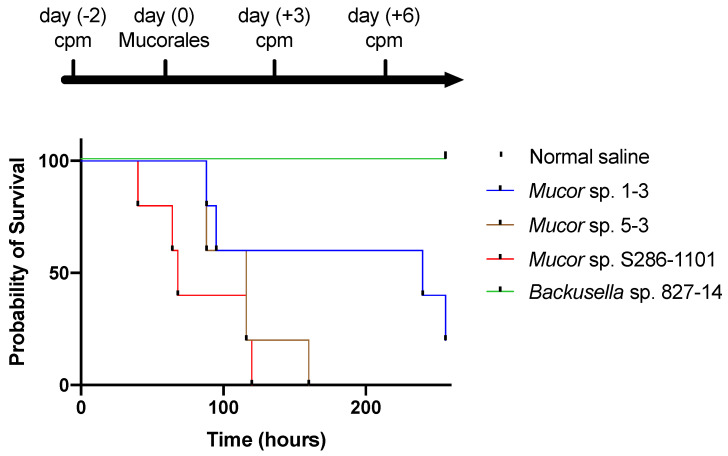
Pathogenicity of Mucorales spores in mice. Four weeks old ICR female mice (n = 5) were immunocompromised by injecting cyclophosphamide (cpm) and injected with 2.0 × 10^8^ colony-forming units of Mucorales spores through the intraperitoneal route and survival was recorded at various time points.

Next, we tested the ability of these strains to infect mammalian hosts using an immunocompromised mouse model. The result showed that three out of four strains could kill the mouse within four days of the infection suggesting their pathogenicity to mammals (Figure 8).

## 4. Discussion

Mucormycosis, previously referred to as zygomycosis, is one of the most common mold infections in immunocompromised patients, caused by a group of fungi belonging to the order Mucorales. There are several genera within Mucorales that are capable of infecting humans, such as *Rhizopus* and *Mucor* [31]. *Rhizopus* and *Mucor* were previously categorized under the Mucoraceae family; however, recent advancements in taxonomic studies have kept them under two separate families Rhizopodaceae and Mucoraceae, respectively [32]. Mucormycosis is characterized by tissue necrosis and vascular invasion and has a high mortality rate. Compared to other opportunistic molds, mucormycosis causing Mucorales are characterized by their ability to infect a broader and more heterogeneous host [33,34]. As they exist in the environment as spores, people develop the risk of infection after inhaling or ingesting the spores or acquiring them through ruptured skin surfaces. Although considered rare previously, in recent days, there has been a tremendous increase in the number of infections [35]. Various mammalian pathogenic fungi are dimorphic in response to temperature, but the fungi that cause mucormycosis are non-thermally dimorphic and show a dimorphic nature in response to O_2_ and CO_2_ tensions [36]. Consistent with this, we found that our *Mucor* strains displayed a dimorphic nature based on O_2_ availability.

Understanding the habitat, physiology, and pathogenicity of Mucorales would provide information that can broaden our knowledge about emerging virulent Mucorales and might help design prevention and treatment strategies for mucormycosis. In this study, we identified four new pathogenic Mucorales from the environment using silkworms, three of which, were pathogenic to mice suggesting the applicability of the silkworm model in identifying fungi pathogenic to mammals. Mucorales are ubiquitously present, highly resistant to commonly used antifungals [37], and can cause opportunistic infections among immunocompromised individuals. With the immunocompromised condition of COVID-19 patients due to prolonged use of corticosteroids, there has been an increase in the incidences of COVID-19 associated mucormycosis. The presence of pathogenic Mucorales in natural environments emphasizes the need for proper preventive and treatment strategies to combat mucormycosis in this situation of the COVID-19 pandemic. Besides, the infectivity of Mucorales to both the silkworms and mice suggested that the silkworm model of mucormycosis could be utilized to identify new pathogenic fungi and their virulence determinants.

## Figures and Tables

**Table 1:** Source and the growth of novel fungi at different temperatures.

Strain	Source (Location)	Growth at		
		37 °C (4 Days)	30 °C (4 Days)	4 °C (10 Days)
*Mucor* sp. 1-3	Plant bud (Chiba, Japan)	−	+++	−
*Mucor* sp. 5-3	Plant seed (Chiba, Japan)	+	+++	−
*Mucor* sp. S286-1101	Plant leaf (Saitama, Japan)	+	+++	−
*Backusella* sp. 827-14	Dead plant leaf (Chiba, Japan)	−	+++	−

+: growth, +++: prominent growth, −: no prominent visible growth.

**Table 2:** General features of *Mucor* sp. 5-3 draft genome.

Features	Characteristics
Total reads	5,016,222
Average length (bp)	270
Coverage	44×
Genome size (bp)	30,813,178
Contigs	17,079
Contigs > 1000 bp	6792
Longest contig (bp)	37,105
*N*_50_	4436
*L*_50_	2075
G + C (%)	39.26

## Data Availability

The sequences of LSU rRNA, ITS region of all the strains and whole-genome sequence of *Mucor* sp. 5-3 have been deposited to NCBI under BioProject PRJNA752231.

## Supplementary Materials

The following are available online at https://www.mdpi.com/article/10.3390/jof7110995/s1, Figure S1: LSU rRNA (a) and ITS (b,c) based phylogenetic analysis of novel Mucorales analyzed using MAFFT and RAxML, Figure S2: Sequence alignment of LSU of newly identified Mucorales, Figure S3. Growth of novel Mucorales, Table S1. Pathogenicity of Mucorales spores in silkworm.
