# Supplementary material for: Novel Pathogenic Mucorales Identified Using the Silkworm Infection Model
Source: J Fungi (Basel). 2021 Nov 22;7(11):995. doi: 10.3390/jof7110995 (PMC8621750; doi:10.3390/jof7110995)
Supplement: Supplementary file 1 [file jof-07-00995-s001.zip › jof-1476576-supplementary.pdf]

Supplementary information to:

Novel pathogenic Mucorales identified using the silkworm infection model

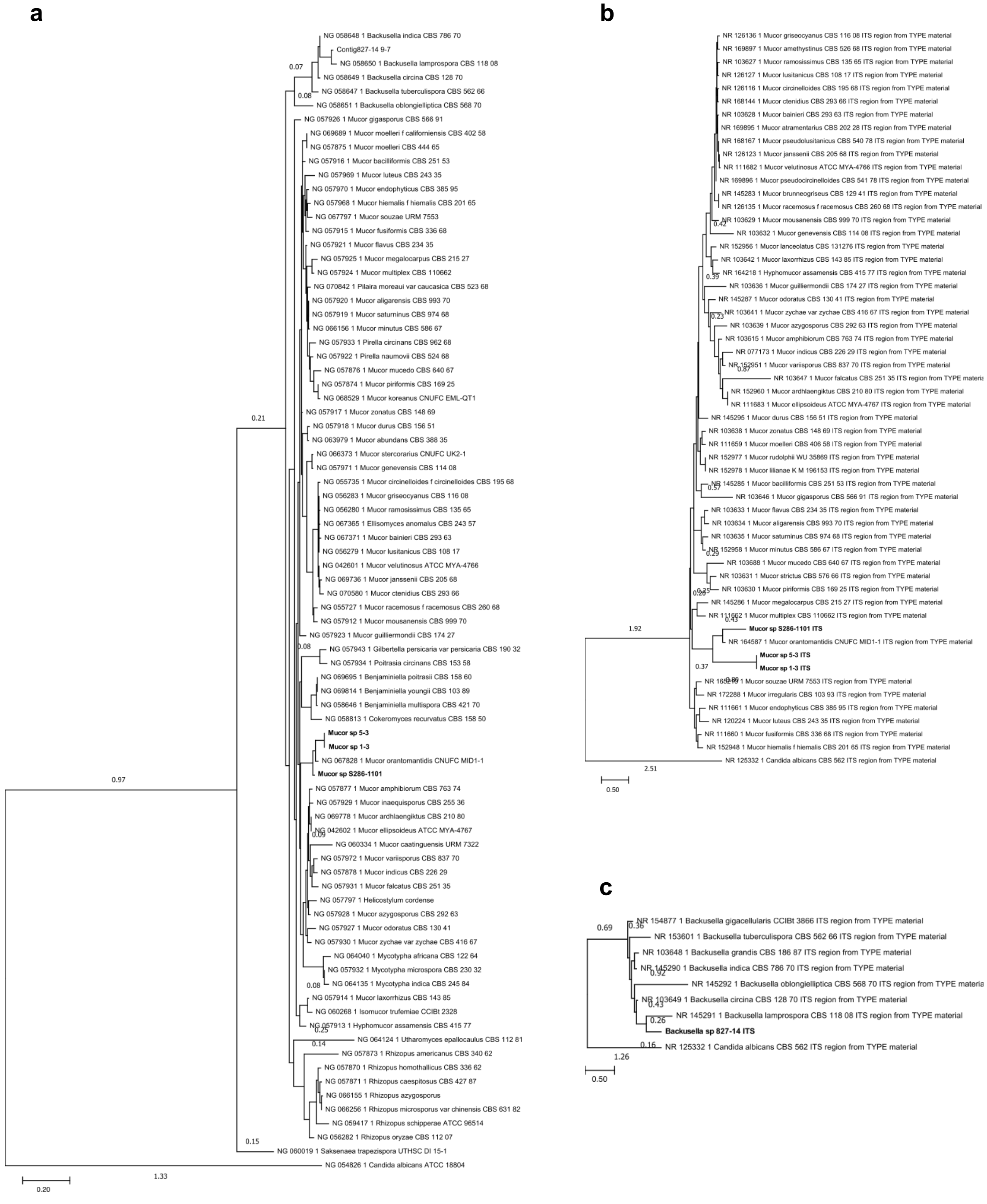

Supplementary Figure S1. LSU rRNA (a) and ITS (b-c) based phylogenetic analysis of novel Mucorales analyzed using MAFFT and RAxML. (a) Both *Mucor* spp and *Backusella* spp (**b**) *Mucor* spp, and (**c**) *Backusella* spp.

```
Backusella sp. 827-14  AAAAGAAAATAACAATGATTTCCCTAGTAACGGCGAGTGAAGAGGAAAGAGCTCAAAGTT  60
Mucor sp. 1-3          AAAAGAAAATAACCATGATTTCCCTAGTAACGGCGAGTGAAGAGGAAAGAGCTCAAAGTT  60
Mucor sp. 5-3          AAAAGAAAATAACCATGATTTCCCTAGTAACGGCGAGTGAAGAGGAAAGAGCTCAAAGTT  60
Mucor sp. S286-1101    AAAAGAAAATAACAATGATTTCCCTAGTAACGGCGAGTGAAGAGGAAAGAGCTCAAAGTT  60
                       ************* **********************************************

Backusella sp. 827-14  GGAATCCGGCTGCCCTAGGTAGTTGGAGTTGTAAACTGGAGAGGTGTTTTCCAGGCGAGC  120
Mucor sp. 1-3          GGAACCTGTTTGGCTTAGCTAAACCGGATTGTAAACTGTAGAGGTGTTTTCCTGACACGC  120
Mucor sp. 5-3          GGAACCTGTTTGGCTTAGCTAAACCGGATTGTAAACTGTAGAGGTGTTTTCCTGACACGC  120
Mucor sp. S286-1101    GGAACCTGTTTGGCTTAGCTAAACCGGATTGTAAACTGTAGAAGTGTTTTCCTGATACGC  120
                       **** * *  ** * *** **    *  ********** *** ********* *    **

Backusella sp. 827-14  CAAGCAAATAAGTCCTTTGGAACGGGGCATCATAGAGGGTGAGAATCCCGTCTTTGGCTT  180
Mucor sp. 1-3          TTGGTAAATAAGTCCTTTGGAACAGGGCATCATAGAGGGTGAGAATCCCGTCTTTGGCCA  180
Mucor sp. 5-3          TTGGTAAATAAGTCCTTTGGAACAGGGCATCATAGAGGGTGAGAATCCCGTCTTTGGCCA  180
Mucor sp. S286-1101    CTGGCAAATAAGTCCTTTGGAACAGGGCATCATAGAGGGTGAGAATCCCGTCTTTGGCCA  180
                          * ****************** **********************************

Backusella sp. 827-14  GAGCTATTCGCCTTTTGCGATACACTTTCAAAGAGTCAGGTTGTTTGGGAATGCAGCCTA  240
Mucor sp. 1-3          AAGCAGT-TGTCTTTTGTGATACACTTTCAAAGAGTCAGGTTGTTTGGGAATGCAGCCTA  239
Mucor sp. 5-3          AAGCAGT-TGTCTTTTGTGATACACTTTCAAAGAGTCAGGTTGTTTGGGAATGCAGCCTA  239
Mucor sp. S286-1101    GAGCAG--TGTCTTTTGTGATACATTTTCAAAGAGTCAGGTTGTTTGGGAATGCAGCCTA  238
                        ***     * ****** ****** ***********************************

Backusella sp. 827-14  AATTGGGTGGTAAATCTCACCTAAAGCTAAATATGGGCGAGAGACCGATAGCGAACAAGT  300
Mucor sp. 1-3          AATTGGGTGGTAAATCTCACCTAAAGCTAAATATCTGCGAGAGACCGATAGCGAACAAGT  299
Mucor sp. 5-3          AATTGGGTGGTAAATCTCACCTAAAGCTAAATATCTGCGAGAGACCGATAGCGAACAAGT  299
Mucor sp. S286-1101    AATTGGGTGGTAAATCTCACCTAAAGCTAAATATCTGCGAGAGACCGATAGCGAACAAGT  298
                       **********************************  ************************

Backusella sp. 827-14  ACCGTGAGGGAAAGATGAAAAGAACTTTGAAAAGAGAGTTAAACAGTATGTGAAATTGTT  360
Mucor sp. 1-3          ACCGTGAGGGAAAGATGAAAAGAACTTTGAAAAGAGAGTTAAACAGTACGTGAAATTGTT  359
Mucor sp. 5-3          ACCGTGAGGGAAAGATGAAAAGAACTTTGAAAAGAGAGTTAAACAGTACGTGAAATTGTT  359
Mucor sp. S286-1101    ACCGTGAGGGAAAGATGAAAAGAACTTTGAAAAGAGAGTTAAACAGTACGTGAAATTGTT  358
                       ************************************************ ***********

Backusella sp. 827-14  GAAAGGGAACCGTTTGGAGCCAGACCGGCCGTTCTATAATCAATCTCTGCCTCGGTGGGG  420
Mucor sp. 1-3          AAAATGGAACGGTTTGAAGCCAGATTGGCTTGGTTGTAATCAATCTAGGATTCGTCCTGG  419
Mucor sp. 5-3          AAAATGGAACGGTTTGAAGCCAGATTGGCTTGGTTGTAATCAATCTAGGATTCGTCCTGG  419
Mucor sp. S286-1101    AAAATGGAACGGTTTGAAGCCAGACTGGCTTGGTTGTAATCAATCTAGGATTCGTCCTGG  418
                        *** ***** ***** *******  ***     * **********  *  ***    **

Backusella sp. 827-14  ATGCACTTGTAGGACGTTGGCCTGCCAACAGCAATTTTGTCTGGTGGAAAAAACCAGGAG  480
Mucor sp. 1-3          ATGCACTTGCAGC--CTATGCCTGCCAACGACAGTTTTTCTTGAGGGAAAAAGCCATTGG  477
Mucor sp. 5-3          ATGCACTTGCAGC--CTATGCCTGCCAACGACAGTTTTTCTTGAGGGAAAAAGCCATTGG  477
Mucor sp. S286-1101    ATGCACTTGCAGC--CTATGCCTGCCAACGACAGTTTTTTCTGAGGGAAAAAACTAGAGG  476
                       ********* **    * **********  ** ****   **  ******* * *   *

Backusella sp. 827-14  GAATGTGGCCCCTCGGGGTGTTATAGCCTCCCGGAAAATACATCGGACGGGATTGAGGAC  540
Mucor sp. 1-3          AAATGTGGCCCTTCGGGGTGTTATAGTCCTTTGGAAAATACCTTGGGAAAGACTGAGGAA  537
Mucor sp. 5-3          AAATGTGGCCCTTCGGGGTGTTATAGTCCTTTGGAAAATACCTTGGGAAAGACTGAGGAA  537
Mucor sp. S286-1101    AAATGTGGCCCTTCGGGGTGTTATAGTCCTTTAGAAAATACCTTGGAAGAGACTGAGGAA  536
                        ********** ************** *     ******** * **    ** ******

Backusella sp. 827-14  TGCAGTAAATGCCTTTAGGCG-AAATACCTGGGCGCTTGTGTTGAGGACGTGTAGAATCT  599
Mucor sp. 1-3          CGCAGTGGATGCCTTTATGGCAAGATTGCTGAGTGCTTTTGCTGATGTATGCTAGAATTT  597
Mucor sp. 5-3          CGCAGTGGATGCCTTTATGGCAAGATTGCTGGGTGCTTTTGCTAATGTATGCTAGAATTT  597
Mucor sp. S286-1101    CGCAGTGAATGCCTTTA-GGCAAGATTGCTGGGCGCTTTTGTTGATACATGCTAGAATTT  595
                        *****  ********* *   * **  *** * **** ** * *       ****** *

Backusella sp. 827-14  CTGCTTCGGGTGGTGCTACGCTCT-AATGTGCAACTCGCCTAGATTATTTTCTATTTGCT  658
Mucor sp. 1-3          CTGCCTTGGGTGGTGCTAGTGTACAAAGGTAAAACTCGCTTGGTATATTTTTCATCCGCT  657
Mucor sp. 5-3          CTGCCTTGGGTGGTGCTAGTGTACAAAGGTAAAACTCGCTTGGTATATTTTTCATCCGCT  657
Mucor sp. S286-1101    CTGCTTCGGGTGGTGCTAGTGTGTAAAGATGGAACTCGTTTAGTATATTTTTCATTCACT  655
                       **** * ***********   *   **  *  ******  * *  ******  **   **

Backusella sp. 827-14  TAGGTTGTTGGCTTAATGACTCTAAATGAC      688
Mucor sp. 1-3          TAGGTTGTTGGCTTAATGACTTTAAATCAC      687
Mucor sp. 5-3          TAGGTTGTTGGCTTAATGACTTTAAATCAC      687
Mucor sp. S286-1101    TAGGTTGTTGGCTTAATGACTTTAAATCAC      685
                       ********************* ***** **
```

Supplementary Figure S2. Sequence alignment of LSU of newly identified Mucorales.

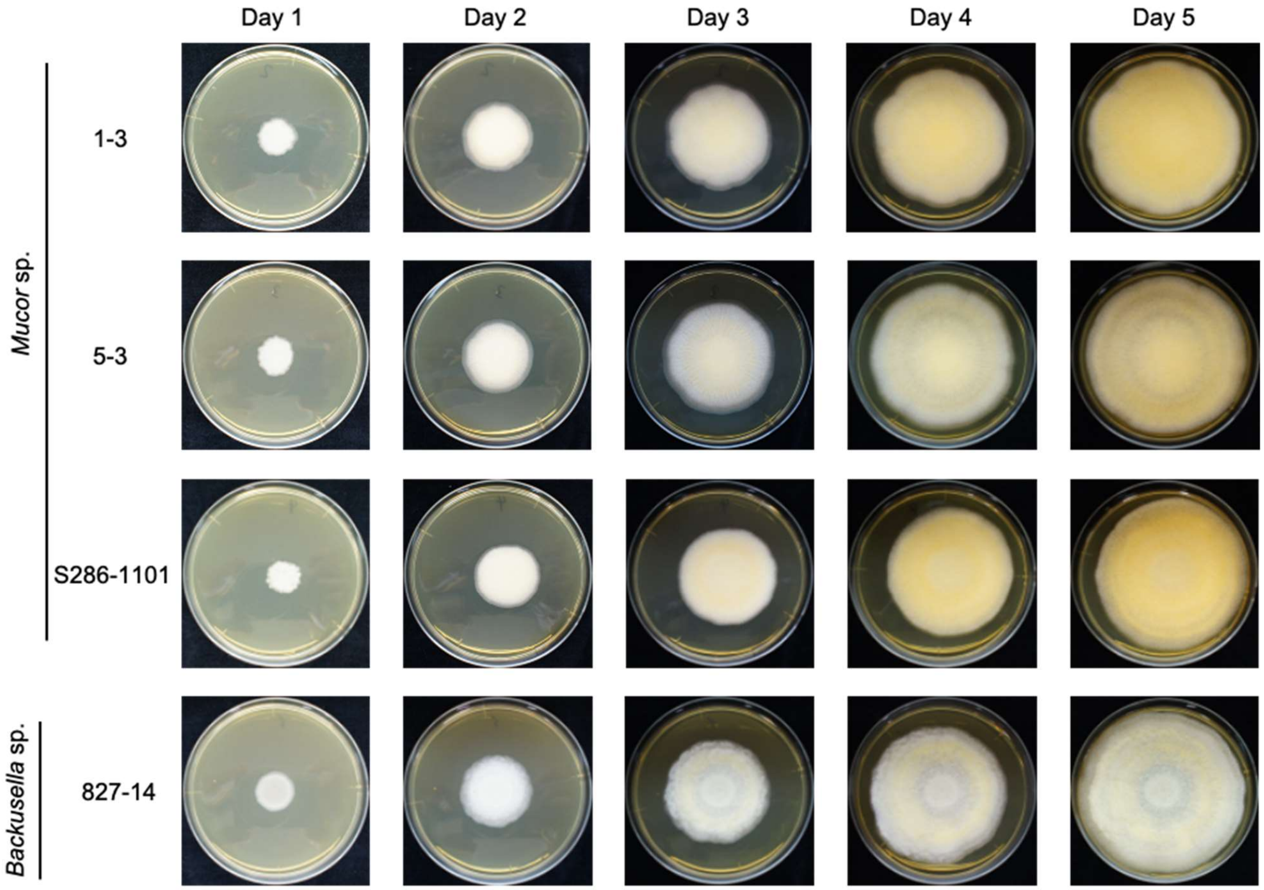

Supplementary Figure S3. Growth of novel Mucorales. 4.0 x 10^4 spores contained in 40 μl of normal saline were spotted at the center of the YPD agar plates, dried, incubated at 30˚C, and growth was recorded every day until the 5th day.

Supplementary Table S1. Pathogenicity of Mucorales spores in silkworm. Fifty microliter spore suspensions with or without 1/8 dilution were injected into the silkworm hemolymph, and survival was recorded at 15 and 20 hrs. NR: Not Recorded

**Sample**	**No of spores/ml**	**Dilution fold**	**Injected silkworms**	**Survival at**	
				15 hr	20 hr
Mucor sp. 1-3	5.5 x 10^8	1	3	NR	0/3
		8	3	2/3	1/3
Mucor sp. 5-3	3.9 x 10^8	1	3	NR	0/3
		8	3	3/3	2/3
Mucor sp. S286-1101	3.5 x 10^8	1	3	NR	0/3
		8	3	3/3	1/3
Backusella sp. 827-14	4.5 x 10^7	1	3	NR	0/3
		8	3	3/3	0/3
Normal saline	0	NA	3	3/3	3/3
